# The association of *COMT* genotype with buproprion treatment response in the treatment of major depressive disorder

**DOI:** 10.1002/brb3.1692

**Published:** 2020-05-27

**Authors:** Jay Fawver, Mindy Flanagan, Thomas Smith, Michelle Drouin, Michael Mirro

**Affiliations:** ^1^ Parkview Health Parkview Physicians Group (PPG) – Mind‐Body Medicine Fort Wayne IN USA; ^2^ Parkview Mirro Center for Research and Innovation Fort Wayne IN USA; ^3^ Manchester University Fort Wayne IN USA; ^4^ Purdue University Fort Wayne Fort Wayne IN USA; ^5^ IUPUI School of Informatics and Computing Indianapolis IN USA; ^6^ Indiana University School of Medicine Indianapolis IN USA

**Keywords:** antidepressants, depression, genetics, pharmacotherapy, treatment

## Abstract

**Background:**

Pharmacodynamics and pharmacogenetics are being explored in pharmacological treatment response for major depressive disorder (MDD). Interactions between genotype and treatment response may be dose dependent. In this study, we examined whether MDD patients with *Met/Met, Met/Val, and Val/Val COMT* genotypes differed in their response to bupropion in terms of depression scores.

**Methods:**

This study utilized a convenience sample of 241 adult outpatients (≥18 years) who met DSM‐5 criteria for MDD and had visits at a Midwest psychopharmacology clinic between February 2016 and January 2017. Exclusion criteria included various comorbid medical, neurological, and psychiatric conditions and current use of benzodiazepines or narcotics. Participants completed genetic testing and the 9 question patient‐rated Patient Health Questionnaire (PHQ‐9) at each clinic visit (*M* = 3.8 visits, *SD* = 1.5) and were prescribed bupropion or another antidepressant drug. All participants were adherent to pharmacotherapy treatment recommendations for >2 months following genetic testing.

**Results:**

Participants were mostly Caucasian (85.9%) outpatients (154 female and 87 male) who were 44.5 years old, on average (*SD* = 17.9). For *Val* carriers, high bupropion doses resulted in significantly lower PHQ‐9 scores than no bupropion (*t*(868) = 5.04, *p *< .001) or low dose bupropion (*t*(868) = 3.29, *p* = .001). *Val* carriers differed significantly from *Met/Met* patients in response to high dose bupropion (*t*(868) = −2.03, *p* = .04), but not to low dose bupropion.

**Conclusion:**

High‐dose bupropion is beneficial for MDD patients with *Met/Val* or *Val/Val COMT* genotypes, but not for patients with *Met/Met* genotype. Prospective studies are necessary to replicate this pharmacodynamic relationship between bupropion and *COMT* genotypes and explore economic and clinical outcomes.

## Graphical abstract

Individuals (154 female and 87 male) with major depression disorder (MDD) with Met/Met, Met/Val, and Val/Val COMT genotypes differed in their response to bupropion treatment in terms of PHQ‐9 scores. High‐dose bupropion is beneficial for MDD patients with Met/Val or Val/Val COMT genotypes, but not for patients with Met/Met genotype.

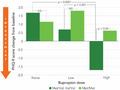

## 1 INTRODUCTION

Major depressive disorder (MDD) is a poorly understood chronic illness characterized by major alterations in mood that, even with antidepressant treatment, can result in significant suicidal ideation leading to death (Madsen et al., 2019). The clinical manifestations of MDD are typically experienced as profound sadness accompanied by numerous physiological changes, such as disturbances in sleep, appetite, sexual desire, constipation and loss of joy and pleasure with friends and co‐workers (Hollon et al., 2006). The lifetime frequency of MDD is approximately 15%, and it is widely accepted that a significant cohort (at least 40%) has a genetic predilection for this disease (Lohoff, 2010). Environmental factors also play a significant role, an epigenetic factor in expressing the phenotypic manifestations of this disease (Nagy, Vaillancourt, & Turecki, 2018).

Given the complexity of genetic and environmental factors, clinicians employ a myriad of treatment interventions with varying levels of success at the individual patient level (e.g., psychotherapy, electroconvulsive therapy, and antidepressants). Pharmacotherapy is a mainstay of modern MDD treatment, but many patients do not respond to initial treatment or discontinue treatment because of adverse drug effects (Trivedi et al., 2006). Although many drugs are licensed for use in MDD, data do not consistently suggest one class or specific medication to be superior in terms of efficacy (Cipriani et al., 2018). Thus, therapeutic options frequently depend upon prescriber familiarity and comfort, patients’ prior experience, cost, and other factors. Despite the discovery and commercialization of new antidepressants, very little work has focused on prospectively characterizing a personalized approach to predicting the pharmacogenetic and pharmacodynamic response to a particular therapy.

The revolution in genomic medicine holds the promise of harnessing genetic data to improve outcomes, increase the likelihood of tolerability, and decrease treatment costs. Pharmacogenetics is one form of personalized medicine involving the use of an individual's genomic profile to help predict optimal treatment outcomes. Emerging data suggest that improved outcomes as well as decreased costs can be obtained in mental illness patients using pharmacogenetics, as it is already doing in disciplines such as oncology and cardiology (Bousman, Arandjelovic, Mancuso, Eyre, & Dunlop, 2019; Perlis, Mehta, Edwards, Tiwari, & Imbens, 2018). Genetic variation is an important factor that influences the efficacy and tolerability (therapeutic index) of pharmaceutical agents, including psychotropic drugs. In fact, many pharmaceuticals, including psychotropic drugs, have biomarker warnings or precautions in their prescribing information with respect to the effect of variants of genes on the drug's exposure. The US Food and Drug Administration (FDA, 2020) notes that “Pharmacogenomics can play an important role in identifying responders and non‐responders to medications, avoiding adverse events, and optimizing drug dose.” Pharmaceutical companies have also begun to state within their prescribing information packets that genotypes can influence dosage and tolerability.

Several commercial pharmacogenetic assays tailored to psychiatry patients are available. The genes for which these assays test include pharmacokinetic (PK) genes and pharmacodynamic (PD) genes. Included PK genes are most often of the CYP450 family, which encode for ubiquitous proteins responsible for the metabolism of most drugs. Two of these PK genes (cytochrome P450 2D6 (CYP2D6) and cytochrome P450 2C19 (CYP2C19)), along with genes involved in hypersensitivity reactions (human leukocyte antigen, B type, allele 15:02 (HLA‐B*15:02) and human leukocyte antigen, A type, allele 31:01 (HLA‐A*31:01)), are currently the four genes that have amassed a level of empirical support to include them in FDA labeling (Miller, 2019).

Pharmacodynamic genes encode for proteins such as transporters, receptors, growth factors, and other targets. Although potentially actionable, they have less research support compared with the aforementioned PK genes. A PD gene incorporated into one such available pharmacogenetic assay is *COMT*, which encodes for catechol‐o‐methyl transferase, an enzyme responsible for the breakdown of dopamine in the frontal lobes. A common variant is a valine to methionine substitution (val158 → met) resulting in decreased capacity of the enzyme to degrade dopamine. Individuals with the *Val/Val* genotype display elevated enzyme activity and increased dopamine degradation; conversely, patients who are *Met/Met* homozygous have reduced enzyme activity and dopamine degradation (Sawa & Snyder, 2002). Because this gene affects synaptic dopamine levels, it is possible that individuals with the various genotypes (*Val/Val*, *Met/Val*, *Met/Met*) at this locus may vary in their response and/or tolerability to dopaminergic drugs.

Bupropion is a widely used antidepressant with a pro‐dopaminergic mechanism of action. Occupancy of dopamine transporter receptors (DAT) by bupropion and its metabolites averaged 26% under conditions of steady‐state oral dosing (150 mg every 12 hr of the sustained‐release (SR) formulation) as determined by positron emission tomography (Learned‐Coughlin et al., 2003). Norepinephrine transporter receptor occupancy has been reported to be similar to DAT occupancy (Masana, Castañé, Santana, Bortolozzi, & Artigas, 2012), possibly suggesting synergism of dopamine and norepinephrine synaptic transmission and therefore not requiring the 80%–90% occupancy required by serotonin receptor transporters. To avoid addictive features, a low level, slow onset, and long‐lasting DAT occupancy is preferable for antidepressant treatment, targeting the phenotype of reduced positive affect symptoms of MDD, including sadness, anhedonia, low energy, and poor motivation (Stahl, 2013).

*COMT* genotyping has been useful in predicting psychostimulant responses for attention deficit disorder (Myer, Boland, & Faraone, 2018). Available genetic testing has been utilized to pharmacodynamically evaluate the association between *COMT* genotypes and bupropion for smoking cessation, but not for the treatment of MDD (Salloum et al., 2018). Considering the biphasic synaptic dopamine levels observed for the *COMT Val/Val* versus *Met/Met* genotypes, we hypothesized that antidepressant response to bupropion would be influenced by the *COMT* genotype, especially in comparing low‐dose versus high‐dose bupropion. This retrospective single‐center study explored the outcomes of patients treated for MDD with pharmacogenomic testing before initiation of treatment.

## 2 MATERIALS AND METHODS

An IRB‐approved retrospective chart review of 241 outpatients was conducted to investigate the correlation of antidepressant effects of bupropion with *COMT* gene variants on individuals who met the DSM‐5 criteria for MDD at various levels of treatment.

Participants were MDD patients at a Midwestern psychopharmacology clinic who had available genetic testing results (Genecept Assay® v. 2.0 [Genomind, Inc.]) conducted between 1 February 2016 and 31 January 2017. As this was a naturalistic study in an outpatient treatment setting, patient treatment plans ranged from initial diagnosis to medication management for those who had failed at least two antidepressants in different classes with an adequate dose and duration. A retrospective chart review utilizing electronic health record data extraction collected the following variables: demographics (age, race), *COMT* variant (rs4680), date of genetic testing, primary and secondary diagnoses, dates of clinic visits that occurred 6 months prior to and 6 months after genetic testing, class and dose of antidepressant medications at time of each clinic visit, and the Patient Health Questionnaire (PHQ‐9) scores at each clinic visit. The items in the PHQ‐9 correspond to the nine symptoms listed in the DSM 5 for an MDD diagnosis. Additionally, participants met the following inclusion criteria: treated with pharmacotherapy; adherent to treatment recommendations based on genetic testing results for >2 months; and 18 years or older at the time of testing. Exclusion criteria included various comorbid medical conditions, current use of benzodiazepines or narcotics; comorbid neurological conditions and various other psychiatric comorbidities. Bupropion doses (almost exclusively in the XL formulation) were categorized as no bupropion, <200 mg (low dose), or ≥200 mg (high dose). *COMT* gene variants were classified as *Met/Val*, *Val/Val*, or *Met/Met*.

Descriptive statistics were calculated to characterize the sample for demographics, *COMT* genetic variants, and bupropion dosing. Four cases had erroneous data for their PHQ‐9 scores and were removed. Chi‐square tests of independence were used to determine whether *COMT* gene variant was related to treatment with bupropion or bupropion dose at time of genetic testing or for new bupropion prescriptions subsequent to genetic testing.

Multilevel models (or linear mixed‐effect models) were estimated. This approach allows integration of the repeated observations for each case, while also incorporating the impact of genetic, demographic, and bupropion predictors (Snijders & Bosker, 2012). One justification for this approach was the high intraclass correlation (42%) observed, suggesting that a high proportion of variance was due to clustering by individual. For the first model, the time sequence of observations was coded as follows: *Pregenetic* represented clinic visits before genetic testing was conducted; *Placebo* represented time period during which genetic testing had occurred, but before results were available and incorporated into the patient medical treatment (set at 4 weeks after genetic testing). For these observations, the simple effect of informing the patients that a genetic test is being conducted can be estimated; this effect was operationalized as changes to PHQ‐9 scores for this time frame. In the statistical analysis, we are thus able to detect a psychological effect simply due to the genetic testing process before the changes to medical treatment, in light of the genetic test results, are implemented.

Secondly, patient demographics, *COMT* gene variants, and bupropion dose were tested for relation to PHQ‐9 scores. Time was also included in these models such that Time 0 represented time period before genetic testing, Time 1 represented visit coinciding with genetic testing, and Times 2, 3,…, *n* represented subsequent clinic visits. According to the primary study hypothesis, *COMT* genetic variant was expected to moderate effects of bupropion dosing on PHQ‐9 scores. Therefore, an interaction term was included in the model. Age and gender were also tested as covariates in all models and retained where significant. Models were specified using Restricted Maximum Likelihood and unstructured covariance and included tests of differences between all combinations of bupropion dose and *COMT* gene variant.

## 3 RESULTS

This study demonstrated high dose bupropion was beneficial for patients with *Met/Val* or *Val/Val* variants, but not for patients with *Met/Met* variants. The total sample included 241 cases with 1,120 observations. On average, participants had 3.8 clinic visits (*SD* = 1.5, range 1–10) from genetic testing date to 6 months after genetic testing. Other sample characteristics, including demographic variables and *COMT* genotype variant, are displayed in Table 1.

**TABLE 1:** Sample characteristics

Characteristic	*N* (%)
Gender	
Female	154/241 (63.9%)
Male	87/241 (36.1%)
Age	
18–29	64/241 (26.6%)
30–39	42/241 (17.4%)
40–49	40/24 (16.6%)
50–59	34/241 (14.1%)
60–69	38/241 (15.8%)
70 and older	23/241 (9.5%)
Race	
White	207/241 (85.9%)
American Indian	1/241 (0.4%)
Black	5/241 (2.1%)
Hispanic	1/241 (0.4%)
Declined/missing	25/241 (10.4%)
*COMT* variant	
*Met/Met*	60/241 (24.9%)
*Val/Met*	129/241 (53.5%)
*Val/Val*	52/241 (21.6%)

The distribution of *COMT* genotypes was typical of a largely Caucasian population. At any time during their treatment from baseline to up to 6 months after genetic testing, medication distributions were noted as 39.0% SSRI, 41.1% SNRI, 32.4% vortioxetine, and 49.4% bupropion.

At the time of genetic testing, 24.1% (*n* = 53) were currently taking bupropion. See Table 2 for percentages of cases taking bupropion and high dose bupropion by *COMT* gene variant. Over the course of the observation period, 16.7% (*n* = 10) *Met/Met*, 22.5% (*n* = 29) *Met/Val*, and 26.9% (*n* = 14) *Val/Val* cases started a new prescription for bupropion.

**TABLE 2:** Sample size, percentage of cases taking bupropion and high dose bupropion at time of genetic testing by *COMT* gene variant (*n* = 220)

*COMT* gene variant	Bupropion, *N* (%)	High dose bupropion, *N* (%)
*Met/Met*	18/58 (31%)	12/58 (21%)
*Val/Met*	24/118 (20.3%)	15/118 (12.7%)
*Val/Val*	11/44 (25%)	6/44 (14%)

Note

Not all cases were observed at Time 1 (genetic testing) so sample size is slightly reduced for this frequency. At time of genetic testing, of those not taking bupropion, 58 cases on SSRI, 23 on SNRI, and 14 on vortioxetine.

In tests of independence between *COMT* gene variant and bupropion, *COMT* gene variant was unrelated to treatment with bupropion and bupropion dose at the time of genetic testing and unrelated to new bupropion prescriptions subsequent to genetic testing. Genetic testing did have a statistically significant effect on PHQ‐9 scores, however, not in the expected direction for a placebo effect. As shown in Table 3, the predicted value of PHQ‐9 was increased by 0.93 units during *Placebo* period.

**TABLE 3:** Summary of multilevel model results for placebo effect on PHQ‐9 scores (*n* = 240)

Fixed effects	Estimate	*SE*	*t* value	*p* > |*t*|
Intercept	13.80	1.06	13.05	<.001
Age	−0.06	0.02	−2.78	.006
Placebo (vs. Pregenetic testing)	0.93	0.46	1.99	.047

Variance components	Estimate	*SE*	*z* value	*p *> |*z*|
Intercept (subject)	21.48	3.07	7.01	<.001
Residual	18.69	1.51	12.35	<.001

As shown in Table 4, time and age were significantly related to reduction in PHQ‐9 scores. Because age was included as a separate predictor, the effect of time is the benefit of treatment visits across all ages. The estimates for *Met/Val* and *Val/Val* indicate that patients in that category had noticeably lower PHQ‐9 scores compared to patients with *Met/Met* gene variant. Also, a significant interaction between bupropion dose and *COMT* gene variant emerged (see Table 5).

**TABLE 4:** Summary from multilevel model predicting PHQ‐9 scores (*n* = 241)

Fixed effects	Parameter estimate	*SE*	*F* value	*p *> |*F*|
Intercept	10.29	1.03	–	–
Age	−0.03	0.02	3.85	.05
Time	−0.70	0.09	64.90	<.001
*COMT* gene variant: Met/Met versus other	2.33	1.15	1.68	.20
Bupropion dose				
Low versus High	3.39	0.67	5.89	.003
Medium versus High	2.40	0.73		
COMT gene variant × Bupropion dose				
Met/Met, Low dose	−2.86	1.17	3.27	.04
Met/Met, Medium dose	−1.22	1.33		

**TABLE 5:** Predicted values and standard errors for PHQ‐9 scores from multilevel model (*n* = 241)

Fixed effects	Predicted value	*SE*	*F* value	*p* > |*F*|
Age				
26.6 (−1 *SD*)	11.82	0.52	3.85	.05
44.5 (mean)	11.23	0.41		
62.4 (+1 *SD*)	10.63	0.50		
Time				
1st visit (genetic testing)	12.00	0.85	64.90	<.001
3rd visit	10.61	0.86		
5th visit	9.21	0.89		
*COMT* gene variant				
*Met/Met*	13.19	1.02	1.68	.20
*Val/Val*, *Met/Val*	12.22	0.87		
Bupropion dose				
None	13.42	0.87	5.89	.003
Low	13.24	0.95		
High	11.45	0.98		
*COMT* gene × Bupropion dose				
*Met/Met* × None	13.15	1.03	3.27	.04
*Met/Met* × Low	13.80	1.23		
*Met/Met* × High	12.62	1.24		
*Val/Val*, *Met/Val* × None	13.68	0.85		
*Val/Val*, *Met/Val* × Low	12.69	0.96		
*Val/Val*, *Met/Val* × High	10.29	1.03		

**FIGURE 1:**
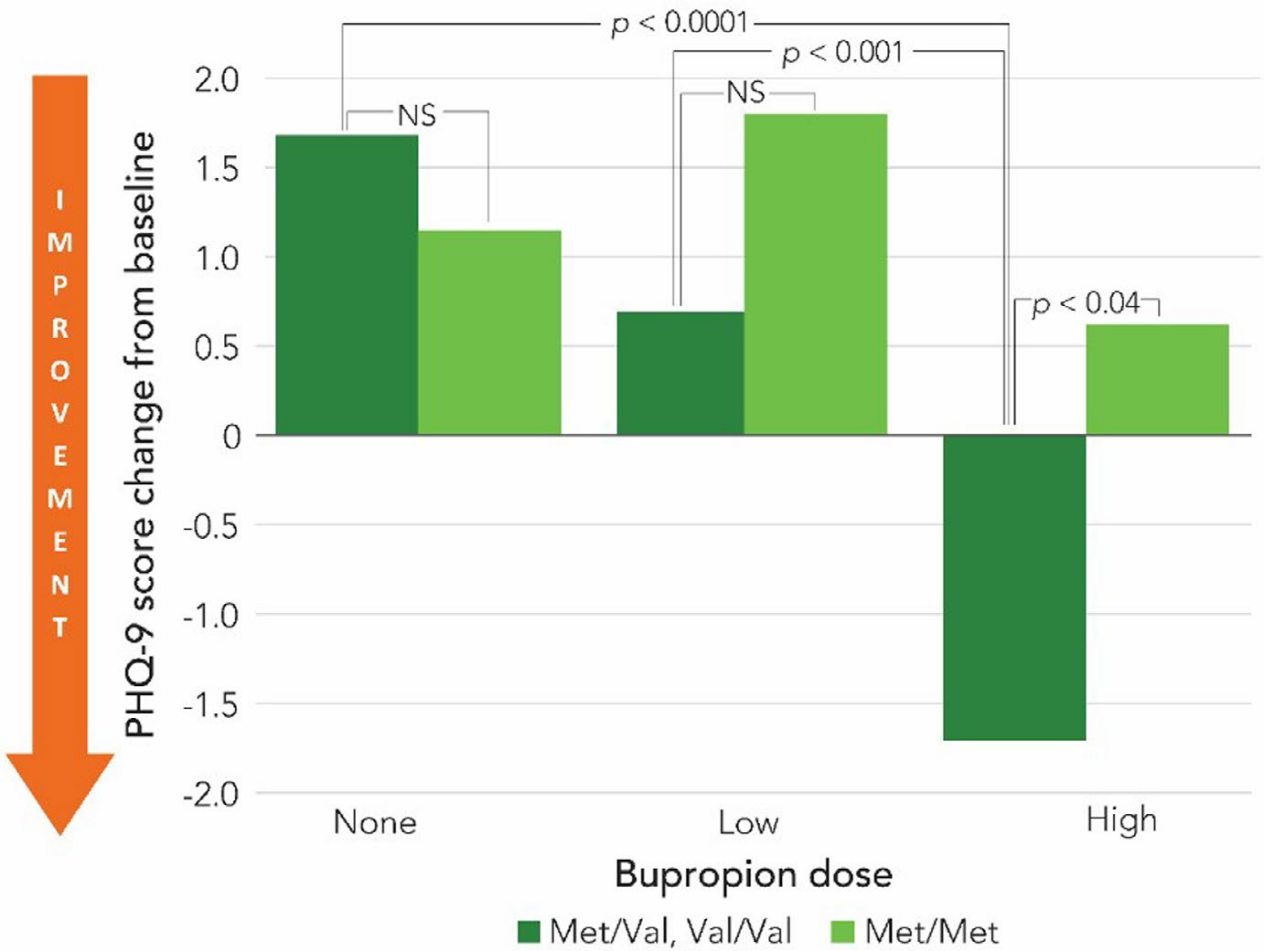
PHQ‐9 predicted values for *COMT* gene variant by Bupropion dose (*n* = 241)

High dose bupropion was beneficial for patients with *Met/Val* or *Val/Val* variants, but not for patients with *Met/Met* variants. This interaction is illustration in Figure 1. Tests for differences in predicted values by level of bupropion dose show that, for individuals with *Met/Met* gene variant, PHQ‐9 scores did not differ by dose. In contrast, for individuals with *Met/Val* or *Val/Val* gene variants, significant declines in PHQ‐9 scores emerged between no bupropion and high dose bupropion (*t*(868) = 5.04, *p *< .001) and between low dose bupropion and high dose bupropion (*t*(868) = 3.29, *p* = .001). Finally, although these two categories of *COMT* genetic variants did not differ significantly in PHQ‐9 scores at low dose bupropion, *Met/Val* or *Val/Val* gene variants did differ significantly in response to high dose bupropion (*t*(868) = −2.03, *p* = .04) compared to *Met/Met* gene variant.

## 4 DISCUSSION

Genetic polymorphisms play a role in the response to medications, an emerging science known as pharmacogenetics. In recent years, commercial pharmacogenetic assays have gained traction in medicine, and data suggest that these assays may be useful in selecting appropriate pharmacotherapy and reducing healthcare costs and utilization (Bousman et al., 2019; Perlis et al., 2018). The FDA’s Table of Pharmacogenomic Biomarkers in Drug Labeling lists at least 260 medications with genomic biomarkers in their label that affect drug exposure and clinical response, risk for adverse events, and genotype‐specific dosing, including several dozen drugs commonly used by mental health clinicians (FDA, 2020). The highest quality data generally are considered to exist for cytochrome p450 gene‐drug associations, but pharmacodynamic gene polymorphisms may also prove useful.

Data suggest that the distribution of cognitive flexibility/rigidity follows a U‐shaped curve, in which too little or too much synaptic dopamine in the frontal cortex can result in impairment (Cools & D’esposito, 2011; Schacht, 2016; Stein et al., 2006). The biphasic nature of the *COMT Val/Val* versus *Met/Met* genotypes has lent itself to studies of outcomes of dopaminergic drugs. Drugs related to dopamine enhancement (amphetamines, methylphenidate, and *COMT* inhibitors) have been associated with greater benefit in *COMT Val/Val* individuals, whereas drugs acting via dopamine antagonism (atypical antipsychotics) have been associated with greater benefit in *Met/Met* carriers (Farrell, Tunbridge, Braeutigam, & Harrison, 2012; Hamidovic, Dlugos, Palmer, & De wit, 2010; Huang et al., 2016; Myer et al., 2018).

Catechol‐O‐methyltransferase (*COMT*) metabolizes catecholamines; thus, the association of *COMT* genotypes in treating MDD has been examined in terms of treatment response for some drugs. For example, two published studies on the role of the *COMT* val158met polymorphism in antidepressant treatment response investigating samples of 102 and 346 patients, respectively, report a tentative negative effect of the *COMT* 158*Met/Met* genotype on mirtazapine and citalopram response in MDD (Arias et al., 2006; Szegedi et al., 2005). Meanwhile, a systematic review and meta‐analysis of pharmacogenetics for MDD, using data from four studies and STAR‐D data, *COMT* was unrelated to antidepressant (SSRI or non‐SSRI) response or remission (Niitsu, Fabbri, Bentini, & Serretti, 2013). Finally, in a previous study of 268 patients and an age‐ and gender‐matched control sample of 557 healthy probands, a negative influence of the higher activity *COMT* 158*Val/Val* genotype on antidepressant treatment response was identified during the first 6 weeks of pharmacological treatment in MDD (Baune et al., 2008). The medications studied were mirtazapine, citalopram/escitalopram, venlafaxine, mirtazapine plus citalopram/escitalopram, mirtazapine plus venlafaxine, tricyclic antidepressants, monoamine oxidase inhibitors, lithium with possible co‐medication with quetiapine, olanzapine, risperidone, lithium, or valproate acid. This compromised treatment response for patients with the *COMT* 158*Val/Val* genotype was conferred by the likelihood of decreased dopamine availability, suggesting a potentially beneficial effect of an antidepressive add‐on therapy with substances increasing dopamine availability individually tailored according to *COMT* val158met genotype (Baune et al., 2008).

Although the association between *COMT* variant and bupropion treatment response for MDD has not been studied previously, bupropion has been examined in terms of its efficacy with different phenotypic symptoms. Most notably, a systematic review and meta‐analysis identified 51 studies, divided into four categories: bupropion as a sole antidepressant, bupropion coprescribed with another antidepressant, bupropion in “other” populations (e.g., bipolar depression and elderly populations), and primary evaluation of side effects (Patel et al., 2016). Some data supported bupropion targeting specific phenotypic symptoms, but insufficient information was available to reliably inform such prescribing. Thus, it remains uncertain whether bupropion pharmacodynamically augments other drugs.

Based on the previous literature, we hypothesized that a specific variant of *COMT*, val158 → met, could affect response to bupropion in patients with MDD. As genomic psychiatry is still in a nascent phase, we first examined whether genetic testing, in itself, had a significant effect on patients’ PHQ‐9 scores. Genetic testing did have a statistically significant effect on PHQ‐9 scores, however, not in the expected direction for a placebo effect—patients’ scores increased significantly in the time between genetic testing and incorporation of these results into patients’ medication plans. Thus, there does not appear to be any psychological benefit, in terms of depression scores, in simply administering genetic tests. However, information garnered from the genetic screening was valuable in predicting MDD patients’ responses to treatment. As the *COMT* gene variant was not the focus of medication administration, *COMT* gene variant type was unrelated to treatment with bupropion and bupropion dose at the time of genetic testing and unrelated to new bupropion prescriptions subsequent to genetic testing. However, our retrospective study found that a high dose of bupropion (≥200 mg daily) was beneficial for MDD patients with Val carrier *COMT* genotypes, but not for patients with a *Met/Met* genotype. This is an important and novel finding, as it contradicts previous studies showing no associations between *COMT* variant and remission or medication response for MDD patients (Mcleod, Fang, Luo, Scott, & Evans, 1994).

As a naturalistic study, several limitations exist. This was a single‐center retrospective study conducted on patients in the Midwestern United States. The population was primarily Caucasian females, so results may not be extrapolated to larger, more diverse populations of patients. Additional limitations include the lack of a clinician‐based assessment outcome metric tool and an antidepressant treatment as usual comparator without genotyping. Self‐assessments may also be biased; however, we have no reason to believe that these biases in self‐report would differ significantly between patients with different *COMT* genotypes. That said, a clinical assessment of depression, by a clinician blind to dosage and *COMT* genotype, would provide additional strength to findings. Additionally, the patients occasionally used combination and adjunctive treatment for depression. Due to our limited sample size, these combinatorial treatments were not controlled for in this study. Future studies with larger cohorts should control for adjunctive medications.

## 5 CONCLUSION

Our data suggest that a high dose of bupropion (≥200 mg daily) is beneficial for MDD patients with *Val* carrier *COMT* genotypes, but not for patients with a *Met/Met* genotype. While prospective studies are necessary to replicate this pharmacodynamic relationship between bupropion and *COMT* genotypes and explore economic and clinical outcomes, we believe that *COMT Val* carriers (75.1% of patients in this study) should be prescribed bupropion at doses ≥200 mg. *Met/Met* carriers (24.9% of patients in this study) should avoid, or cautiously use bupropion for MDD. The use of genetic testing, although not deterministic, may influence the probability of successful bupropion antidepressant response, especially when considered as a factor combined with depressive phenotyping and past individual and family of antidepressant treatment responses.

Prospective work on the effectiveness of pharmacotherapy for MDD should include an analysis of pharmacodynamics and pharmacogenetic genotypes. This work provides the foundational elements to design a large randomized clinical trial to link the utility of pharmacogenomic testing guided drug selection to clinical outcomes.

## CONFLICT OF INTEREST

Dr. Fawver reports personal fees from Takeda Pharmaceutical Company, Lundbeck, Inc., personal fees from Janssen Pharmaceuticals, Inc., personal fees from Alkermes, outside the submitted work. Dr. Mirro reports grants from Medtronic plc, during the conduct of the study; grants from Agency for Healthcare Research and Quality (AHRQ), grants from Biotronik, Inc, grants from Janssen Scientific Affairs, personal fees from McKesson Corporation, personal fees from iRhythm Technologies, Inc., personal fees from Zoll Medical Corporation, other from Medical Informatics Engineering, outside the submitted work; and Dr. Michael J. Mirro's relationships with academia include serving as trustee of Indiana University and on the Indiana University Health Board.

## AUTHOR CONTRIBUTION

Jay Fawver and Mindy Flanagan helped to conceptualize, design, and conduct this study. Jay Fawver, Michelle Drouin, Mindy Flanagan, Thomas Smith, and Michael Mirro collected, analyzed, and/or interpreted the data and wrote the manuscript. Jay Fawver and Michael Mirro were in charge of overall direction and planning of the project. Additionally, all authors drafted or revised this manuscript critically for important intellectual content, approved the version to be published, and agree to be accountable for all aspects of the work in ensuring that questions related to the accuracy or integrity of any part of the work are appropriately investigated and resolved.

## Data Availability

The data that support the findings of this study are available on request from the corresponding author. The data are not publicly available due to privacy or ethical restrictions.
